# The Australian Nursing Service in the War

**Published:** 1918-05-04

**Authors:** 


### THE AUSTRALIAN NURSING SERVICE IN THE WAR.

When it is remembered that the total population of Australia does not exceed that of Greater London, the sacrifice which that country has made in dispatching 1,700 trained nurses to nurse the wounded may be appreciated. For the last two years they have been playing their part, a very strenuous and efficient part, in Imperial military nursing, and their numbers are by no means yet at their maximum. There is a large reserve still at home from which drafts of forty, fifty, or sixty at a time come over as cabled for. Their Matron-in-Chief is Miss E. A. Conyers, who holds a rank equivalent to that of major. Other matrons hold the rank of captain, sisters of lieutenant, and staff nurses of second lieutenant. The outdoor uniform consists of a dark-grey Norfolk jacket and skirt, with copper buttons and stars according to rank. The No. 2 Australian Auxiliary Hospital at Southall forms the headquarters of the nursing staff in England. There they arrive on reaching England, to receive at once fourteen days' leave, and from thence they are sent on to their next destination, a due number remaining to staff this hospital in the meanwhile. There is a permanent Sea Transport Section, consisting of matron, two sisters, and four staff nurses. The nurses all engage "for the duration," but in case of disabling illness or urgent family affairs are not debarred from returning home. A beautifully equipped little hospital with twenty-five beds has been fitted up for sick nurses of the Force in Southwell Gardens, South Kensington, the gift of Mrs. T. S. Hall, and this is supplemented by Mr. McIlwraith's loan of his son's house Glenalmond at St. Albans as a convalescent home for Australian nurses.

It is a special feature of Australian nursing that one day off duty in seven is the rule. Three hours off duty are allowed every day, or, under circumstances which render this desirable, nurses may remain on duty all one day and get a half-day off the next. In Egypt this was a privilege much appreciated by the staff, as it enabled them to make expeditions. After every six months they get a fortnight's leave.

The Australian contingent has done invaluable service in relieving British nurses whenever they have been needed. Sometimes they have undertaken, when required, the complete staffing of British hospitals abroad; and at other times they have supplemented a hard-pressed British staff. But the principal work undertaken by the Australians has naturally been the staffing of the hospitals provided and equipped by the Australian Government. The first of these to be started was the famous Australian hospital in Bombay. The Australian Commonwealth has now generously equipped and supported numerous hospitals situated in various localities in England, France, and Salonika, as well as those in Egypt and Bombay: all of these are staffed by nurses from the Dominions.

The high standard of training among Australian nurses, their *esprit de corps*, energy, and true comradeship, have made them valued and respected wherever they have been sent.

Among those who have gained distinction during the last few months may be mentioned Sister Estelle Keogh, R.R.C., Sister Nancy Wilder, mentioned in dispatches, Sister Rose King, and Sister Rachel Pratt, who, with three other Australian nurses, received the Military Medal for bravery under fire. The last-named lady was wounded whilst on duty.

☛ For The Making of a Trained Nurse, Possible Changes due to the War, see p. 88; Rations in Hospitals, a New and Authoritative Diet Scale, p. 96; Editor's Letter Box, p. 101; The Economics of Nursing, p. 102; Matrons' and Sisters' Appointments, p. iv.

